# One-week recall period gives a more accurate estimate of exclusive breastfeeding practice than 24-h recall among infants younger than six months of age

**DOI:** 10.1186/s13006-021-00411-2

**Published:** 2021-08-28

**Authors:** Sewitemariam Desalegn Andarge, Esete Habtemariam Fenta, Seifu Hagos Gebreyesus, Robel Yirgu Belachew

**Affiliations:** 1 Department of Reproductive Health and Health Service Management, School of Public Health, College of Health Sciences, Addis Ababa University, Addis Ababa, Ethiopia; 2 Department of Global Health & Infection, Brighton and Sussex Medical School, Brighton, UK

**Keywords:** Exclusive breastfeeding, Accuracy, 24-h recall, Week recall, Overestimation

## Abstract

**Background:**

The World Health Organization recommends a 24-h recall period to estimate breastfeeding practice of mothers of infants aged younger than six-months. Though 24-h recall was preferred for its low recall bias and for practical reasons, it can overestimate exclusive breastfeeding practice (EBF). Validating this indicator will help account for the deviation from the true estimate. This prospective cohort study measured accuracy of the 24-h recall method and validates a week recall as an alternative approach for use in a small sample population.

**Method:**

The study was conducted from March to April 2018 involving 408 mother-infant pairs living in Butajira Health and Demographic Surveillance Site (HDSS), Southern Ethiopia. Participants were prospectively followed for 14 consecutive days; where their breastfeeding practice in the past 24 h was measured daily. Exclusive breastfeeding prevalence estimate obtained using the 24-h recall method and recall periods spanning a varying number of days (short period recalls) was compared against the cumulative of the responses from a prospectively measured repeated 24-h recalls over the course of 14 days. McNemar statistics was used to assess statistical significance of the difference in the EBF prevalence estimates of the single 24-h recall and the reference standard. Sensitivity, specificity, positive predictive value and negative predictive values were calculated to determine the level of accuracy. Receiver Operating Characteristics curve was used to measure the difference in performance between the two methods.

**Result:**

The highest prevalence (71.4%) of exclusive breastfeeding practice was estimated using the single 24-h recall method whereas the lowest breastfeeding practice (47.1%) was obtained from a cumulative of 14 repeated 24-h recalls. A week recall (a recall over 7 days’ period), resulted in the smallest discrepancy in estimate (7.1%) as compared to cumulative estimate of 14 repeated 24-h recalls. Comparing against our reference standard, a week recall had 96.7% sensitivity and 83.5% specificity in estimating exclusive breastfeeding practice.

**Conclusions:**

Using single 24-h recall method overestimated exclusive breastfeeding prevalence. However, a week recall gave an estimate close to the estimate from the standard method. A week recall has a potential to balance the tradeoff between the accuracy of EBF estimates and the resource implication of using multiple prospective measurements that have a proven superior accuracy.

## Background

Exclusive breastfeeding (EBF) is an important indicator of infant feeding behavior [1]. World Health Organization (WHO) defines EBF as feeding an infant only with breast milk excluding other liquids, semisolid or solid foods, except medicines, ORS, vitamin and minerals [2]. The WHO stress the importance of breastfeeding and proposed a set of criteria and indicators to measure infant feeding practices [3]. These indicators are used to identify high risk groups for malnutrition, compare the adequacy of infant feeding practice nationally and internationally, monitor and evaluate nutritional interventions and programs and for research purposes [4]. Infant and young child feeding (IYCF) indicators is a group name given to a set of indicators measuring breastfeeding and other young child feeding practices, and “the proportion of infants less than six months of age who are exclusively breastfed in the past 24 hours” is a key indicator measuring breastfeeding practices [5]. It draws on data collected using the 24-h recall method, where mothers are asked to report their child’s feeding practice of the previous day [6].

There are practical justifications to use a one-day recall as a proxy to determine breastfeeding practice. Resource implications of taking repeated measurements, ease of the indicator to make cross country comparisons, and reduced risk of recall bias are among the reasons [7]. Nonetheless, the low accuracy of using a 24-h recall to describe typical dietary pattern of specially a smaller sample is evident. Failing to account for feeding practices beyond the last 24-h could lead to misclassifying infants as exclusively breastfed [8].

Exclusive breastfeeding prevalence estimates obtained using the 24-h recall method often overestimated the true prevalence [9–12]. Studies indicated a 9.2 and 47.4% overestimation when breastfeeding practice data collected using single 24-h recall was compared with prospectively collected data [13, 14]. In our former publication, we have identified a 23.5% overestimation of the prevalence of EBF practice when estimates from single 24-h recall was compared with the cumulative score of seven repeated 24-h recalls [12]. Though seven repeated 24-h recall is more accurate, it is not practical for routine use.

Having a more accurate indicator is important to show the true picture of the practice thereby informing policy makers to take appropriate actions towards improving child feeding practices. In this study we hypothesized that estimates of breastfeeding practice employing a one week recall period has a superior accuracy than the estimates obtained using single 24-h recall. The validation involved comparing the estimates from a recall of breastfeeding practice over a period of one week against the reference standard (cumulative of the estimates from multiple 24-h recall of 14 consecutive days) among infants younger than six months of age.

## Methods

### Study area and period

The study was conducted in a Health and Demographic Surveillance Site (HDSS) in Gurage Zone, Southern Ethiopia. The HDSS was established and is under the School of Public Health, Addis Ababa University. It encompasses one urban and nine rural *kebeles* (i.e. the lowest administrative unit comprising an average of 3000 to 5000 people) [15]. The *Kebeles* were selected from the adjoining Meskan, Mareko and part of Silte Districts of the Zone. The study was conducted from March to April 2018.

### Study design

A community based longitudinal study design was employed.

### Sample size and sampling procedure

The sample size was 408 mother-infant pairs. It was powered to detect a 2% difference in specificity between the contender and reference test, with a 95% level of confidence and 80% power. It was calculated using the sample size estimation formula for validation studies [16].

The Butajira HDSS field staff made regular visits to households under surveillance to collect data on demographic events. The demographic data from their last visit was used as a sampling frame to identify women who gave birth in the past six months from the time of the data collection. The sampling frame included information on the child’s age, sex and name of parents and household contact address. The sampling frame included people under surveillance from all the ten kebeles and we employed simple random sampling technique to select sample participants from the frame.

### Data collection process

A three-day training was given to data collectors, the field staff of the HDSS, before commencing data collection. Interview questionnaire used in the Ethiopian Demographic and Health Survey (EDHS) was adapted to collect data about the socio-economic and demographic characteristics of participants [17]. Breastfeeding practice was measured using the WHO’s itemized check list designed to assess breastfeeding practice [2].

Demographics of the study sample and data on the socio-economic characteristics of their respective households were collected at baseline. Mothers with children of age six months and younger were interviewed about their breastfeeding practices of the previous day and each day for next 14 consecutive days.

### Breastfeeding practice measurement

#### 24 h recall

Mothers were asked to recall their feeding history over the previous 24-h. 24-h recall measurements were conducted/ repeated in each one of the subsequent 14 days.

#### Short recall

In addition to the 24-h recall, we have used short recalls to collect breastfeeding history. Short recalls involved a recall period encompassing successively increasing days between the baseline and the 14th day of data collection. For example, on the second day of the data collection, mothers were asked both about their feeding practice of the past 24-h and the previous two days. Similarly, at the third day of data collection, mothers were asked to recall their breastfeeding practice for the previous 24 h and for the previous three days. This went on until the last day of the data collection where mothers were asked about their feeding practices of the last 24-h and over the past two weeks.

### Data analysis procedure

Data entry and analysis was conducted using EpiData *version 3.2* software and Stata *version-14* statistical software, respectively. Frequency and percentage were calculated for each categorical variable and the mean value of data from continuous variables was estimated.

Households of the study participants were categorized into wealth quintiles based on possession of fixed assets by using principal component analysis a data reduction technique.

Exclusive breastfeeding was calculated by dividing the number of infants younger than six months of age who only received breast milk with no addition of other liquids or foods except oral rehydration salt, vitamins and other medications in the previous day (24-h) to all infants younger than 6-months of age. Age specific EBF prevalence rates were also calculated for those in the range of 0 to1 month, 2 to 3 months, and 4 to 5 months. In this case, the denominator was the total number of infants in the age category.

#### Single 24-h recall

The itemized checklist that was used to assess EBF practice employed entering binary values (i.e, 0 or 1) against the foods and fluids listed out in the checklist. One was entered if a child was fed one of the foods and fluids and zero if a child was not fed any. If a child had not been given any food/fluid listed out in the checklist a child will have a cumulative score of zero indicating the child was exclusively breastfed in the past 24-h. If the value given to a minimum of one item was different from zero then the child was classified as non-exclusively breastfed.

#### Short recall

Breastfeeding practice data that was captured by short recalls (a recall over the number of days the participant stayed in follow-up at the time of the interview) was determined in the same manner. Like that of 24-h recall, binary values of 1 and 0 was given for the list of foods and fluids the child might have received. If the mother’s answer was 0 for all foods or fluids from the list over the recall period, then we classified the child as exclusively breastfed, and as non-exclusive if the score was other than zero. Infants aged less than six months who were fed breast milk only in the stated recall period was the numerator to calculate EBF prevalence in the recall period categories exceeding the last 24-h.

### Reference method

A cumulative score of 14 repeated 24-h recalls was the reference standard. Data for the reference standard was obtained by considering the EBF status of the participants in each one of the 14 separate 24-h recall measurements while the participant was on follow-up. Similarly, a binary value of 1 and 0 was given for the list of foods and fluids the child might have received. If the sum of this list for 14 consecutive 24- h recall was zero then infants were classified as being exclusively breastfeeding and they were classified as being non-exclusively breastfeeding if the sum was different from zero.

The McNemar statistics was employed to test significance of the difference between the standard measurement and the three contender measures of breastfeeding practice (i.e. single 24-h recall, a week recall or short recalls). For all tests statistical significance was set at a *P*-value of 0.05.

In this study sensitivity was a measure of a method to identify infants who were exclusively breastfed. The ability of the method to exclude those who were not exclusively breastfed was the specificity. Positive predictive value and negative predictive values were also computed to evaluate the performances of the methods against the reference standard. Receiver Operating Characteristics curve was employed to compare the performance of different methods.

## Results

From the cohort of 408 mother-infant pairs recruited at the beginning of the follow-up, 391 (95.8%) participated throughout the follow-up period. Sex of the study participants was comparable where 181 (46.3%) infants were male and 210 (53.7%) were females. The mean age of infants was three months (± 1.5SD), 36.5% of the participants cannot read or write and the majority, 315 (80.6%) were housewives (Table 1).

**Table 1:** Sociodemographic characteristics of mothers/care takers and infants Butajira HDSS, Ethiopia, 2018

Socio-economic variables	Frequency	Percentage (%)
I**nfant’s sex**		
Male	181	46.3
Female	210	53.7
**Infant’s age**		
0-1 month	125	31.9
2-3 months	127	32.4
4-5 months	139	35.5
**Mother’s age-**		
15–19	11	2.8
20–24	90	23
25–29	149	38
30–34	81	20.7
35–39	47	12
40–44	13	3.3
**Marital status**		
Single	24	6.1
Married	360	92.1
Separated	7	1.8
**Education**		
Illiterate	144	36.5
Read & write	4	1.0
Primary(1–8)	161	41.1
Secondary(9–12)	60	24.3
Higher*	22	8.9
**Occupation**		
Housewife	315	80.6
Merchant	32	8.2
Government employee	17	4.4
Farmer	27	6.9
**Wealth index**		
Lowest	79	20.2
Low	79	20.2
Middle	78	19.9
High	77	19.6
Highest	78	19.9

Higher * includes higher education and technical and vocational education

Table 2 shows exclusive breastfeeding prevalence estimated by using single 24-h recall and multiple 24-h recall. This multiple 24-h recall is the cumulative score of EBF practice derived from more than one day (two and above consecutive days) interview*.* The highest prevalence was found from single 24-h recall, 71.4% (95% CI 66.9, 75.9) and the second highest estimate was from two consecutive 24 h recall 62.7% (95% CI 57.7, 67.3). The results showed that as the number of repeated 24-h recall increased the prevalence of EBF decreased. The fourteen repeated 24-h recalls resulted in the lowest EBF prevalence, 47.1% (95% CI 42.1, 52.1).

**Table 2:** Exclusive breastfeeding (EBF) prevalence among infants aged = < 6 months using single 24 h recall and *multiple 24 h recalls, Butajira, Ethiopia, (*n* = 391)

Number of 24 h recalls	Frequency	EBF percentage (95% CI)
**Single 24 h recall**	279	71.4 (66.9, 75.9)
**2 consecutive 24 h recalls**	245	62.7 (57.7, 67.3)
**3 consecutive 24 h recalls**	220	56.3 (51.3, 61.1)
**4 consecutive 24 h recalls**	208	53.2 (48.2, 58.1)
**5 consecutive 24 h recalls**	205	52.4 (47.5, 57.4)
**6 consecutive 24 h recalls**	199	50.9 (45.9, 55.8)
**7 consecutive 24 h recalls**	196	50.1 (45.2, 55.1)
**8 consecutive 24 h recalls**	193	49.4 (44.4, 54.3)
**9 consecutive 24 h recalls**	190	48.6 (43.6, 53.5)
**10 consecutive 24 h recalls**	190	48.6 (43.6, 53.5)
**11 consecutive 24 h recalls**	190	48.6 (43.6, 53.5)
**12 consecutive 24 h recalls**	188	48.1 (43.1, 53.1)
**13 consecutive 24 h recalls**	186	47.6 (42.6, 52.5)
**14 consecutive 24 h recalls**	185	47.1 (42.1, 52.1)

*multiple 24 h recall- implies the combined measure of EBF practice derived from more than one day (two and above consecutive days) interview

Based on the data from the short period recalls, the prevalence of exclusive breastfeeding decreased as the duration of recall increased until a recall of the past seven days. After the 7th day recall the estimate in the prevalence of EBF started to increase. A recall of the feeding practice over the past two days yielded an estimated 64.2% (95% CI 59.4, 68.9) prevalence of EBF, but it decreased to 54.2% (95% CI 49.3, 59.2) when the recall period covered the previous seven days (one-week) (Table 3).

**Table 3:** Patterns of changes in estimates of exclusive breastfeeding prevalence among infants aged = < 6 months using 24-h recall and short period recalls by using 14 repeated 24-h recalls as *reference, Butajira, Ethiopia, (*n* = 391)

Number of days to recall	EBF percentage (95% CI)	% of overestimation (95% CI)	McNemar’s ***P***-value
**24 h recall**	71.4 (66.9, 75.9)	24.3 (19.8, 28.8)	< 0.0001*
**2 days recall**	64.2 (59.4, 68.9)	17.1 (13.1, 21.1)	< 0.0001*
**3 days recall**	58.1 (53.1, 62.9)	10.9 (7.2, 14.8)	< 0.0001*
**4 days recall**	57.0 (52.1, 61.9)	9.9 (6.5, 13.4)	< 0.0001*
**5 days recall**	56.3 (51.3, 61.2)	9.1 (5.7, 12.7)	< 0.0001*
**6 days recall**	54.5 (49.5, 59.4)	7.4 (3.9, 10.9)	< 0.0001*
**7 days recall**	54.2 (49.3, 59.2)	7.1 (3.8, 10.5)	< 0.0001*
**8 days recall**	55.8 (50.8, 60.7)	8.6 (5.1, 12.3)	< 0.0001*
**9 days recall**	57.3 (52.3, 62.2)	10.2 (6.5, 13.9)	< 0.0001*
**10 days recall**	60.4 (55.5, 65.2)	13.1 (9.1, 17.5)	< 0.0001*
**11 days recall**	61.9 (57.1, 66.7)	14.8 (10.5, 19.1)	< 0.0001*
**12 days recall**	61.9 (57.1, 66.7)	14.8 (10.6, 19.1)	< 0.0001*
**13 days recall**	62.4 (57.6, 67.2)	15.31 (11.1, 19.5)	< 0.0001*
**14 days recall**	63.9 (59.2, 68.7)	16.8 (12.4, 21.3)	< 0.0001*

*McNemar’s test *p* value < 0.05

*reference - EBF percentage 95% CI of 47.1 (42.1, 52.1)

Column 2 shows the result of short period recall which is a single recall with a period of recall indicated in column 1

A significant difference in exclusive breastfeeding prevalence was observed between the data from short recalls and the reference standard. The highest overestimation, 24.3% (95% CI 19.8, 28.8) was observed when single 24-h recall was employed (Table 3). The overestimation grew smaller when shorter recalls were considered. A recall within a period of one week bear the lowest overestimation, 7.1% (95% CI 3.8, 10.5), but when the recall period exceeded seven days period the over estimation increased.

Table 4 shows change in the patterns of EBF prevalence. The difference in exclusive breastfeeding prevalence was examined among the different age groups. The proportion of infants who were exclusively breastfed decreased as the age of infants increased regardless of the method used. Using short period recalls the prevalence of EBF ranged from 74.4–88.0% among infants aged 0–1 months, from 60.6–81.9% among infants aged 2–3 months and from 29.5–46.7% among infants aged 4–5 months.

**Table 4:** Patterns of changes in estimate of exclusive breastfeeding prevalence among infants aged = < 6 months using *short period recalls and 24-h recall by using 14 repeated 24-h recalls as *reference among different age groups, Butajira, Ethiopia, 2018

Age group (0–1 month(***n*** = 125)			
Recall period	EBF percent (95% CI)	% overestimation (95% CI)	McNemar test
**24 h recall**	88.0 (80.9, 92.6)	20.0 (12.2, 27.8)	< 0.0001*
**2 days recall**	80.8 (72.8, 86.8)	12.8 (6.1, 19.4)	< 0.0001*
**3 days recall**	75.2 (67.6, 82.1)	7.2 (0.8, 13.5)	< 0.0126*
**4 days recall**	74.4 (65.9, 81.3)	6.4 (0.7, 12.0)	< 0.0114*
**5 days recall**	74.4 (65.9, 81.3)	6.4 (0.7, 12.2)	< 0.0114*
**6 days recall**	74.4 (65.9, 81.3)	6.4 (0.7, 12.0)	< 0.0114*
**7 days recall**	75.2 (67.6, 82.7)	7.2 (0.8, 13.5)	< 0.0126*
**8 days recall**	76.8 (68.4, 83.4)	8.8 (2.1, 15.4)	< 0.0045*
**9 days recall**	76.8 (68.4, 83.4)	8.8 (2.1, 15.8)	< 0.0076*
**10 days recall**	80.0 (71.9, 86.1)	12.0 (4.3, 19.6)	< 0.0011*
**11 days recall**	79.2 (71.1, 85.5)	11.2 (3.6, 18.7)	< 0.0017*
**12 days recall**	78.4 (70.2, 84.8)	10.4 (2.6, 18.1)	< 0.0046*
**13 days recall**	84.0 (76.3, 89.5)	16.0 (8.7, 23.2)	< 0.0001*
**14 days recall**	85.6 (74.5, 88.1)	17.6 (9.7, 25.4)	< 0.0001*
**Age group (2–3 month (*n*** **= 127)**			
**Recall period**	**EBF percent (95% CI)**	**% overestimation (95% CI)**	**McNemar test**
**24 h recall**	81.9 (12.2, 25.9)	28.3 (19.7, 36.9)	< 0.0001*
**2 days recall**	71.6 (63.1, 78.9)	17.3 (9.6, 25.0)	< 0.0001*
**3 days recall**	66.1 (57.3, 73.9)	12.5 (5.6, 19.6)	< 0.0001*
**4 days recall**	64.6 (55.7, 72.5)	11.0 (4.3, 17.7)	< 0.0005*
**5 days recall**	64.6 (55.7, 72.5)	11.0 (3.9, 18.1)	< 0.0013*
**6 days recall**	61.4 (52.6, 69.5)	7.8 (1.1, 14.7)	< 0.0213*
**7 days recall**	60.6 (51.7, 68.8)	7.1 (0.8, 13.3)	< 0.0225*
**8 days recall**	62.2 (53.3, 70.3)	8.6 (2.1, 15.2)	< 0.0074*
**9 days recall**	67.7 (58.9, 75.3)	14.1 (7.3, 21.1)	< 0.0001*
**10 days recall**	66.9 (58.2, 74.6)	13.4 (5.9, 20.9)	< 0.0002*
**11 days recall**	69.3 (60.6, 76.7)	15.7 (7.9, 23.6)	< 0.0001*
**12 days recall**	66.9 (58.1, 74.6)	13.4 (5.9, 20.9)	< 0.0002*
**13 days recall**	66.1 (57.4, 73.9)	12.6 (5.2, 19.9)	< 0.0004*
**14 days recall**	70.8 (59.8, 76.1)	17.3 (9.3, 25.4)	< 0.0001*
**Age group (4–5 month (*n*** **= 139)**			
**Recall period**	**EBF percent (95% CI)**	**% overestimation (95% CI)**	**McNemar test**
**24 h recall**	46.7 (44.8, 61.4)	24.4 (16.6, 32.3)	< 0.0001*
**2 days recall**	42.5 (34.4, 50.9)	20.2 (12.7, 27.5)	< 0.0001*
**3 days recall**	35.3 (27.7, 43.6)	12.9 (5.6, 20.2)	< 0.0002*
**4 days recall**	34.5 (27.0, 42.9)	12.2 (6.1, 18.4)	< 0.0001*
**5 days recall**	32.4 (25, 40.7)	10.1 (3.6, 16.5)	< 0.0010*
**6 days recall**	30.2 (23.1, 38.5)	7.9 (1.19, 14.6)	< 0.0192*
**7 days recall**	29.5 (22.4, 37.8)	7.2 (1.13, 13.1)	< 0.0129*
**8 days recall**	30.9 (23.7, 39.2)	8.6 (2.1, 15.1)	< 0.0075*
**9 days recall**	30.2 (23.1, 38.5)	7.9 (1.5, 14.3)	< 0.0127*
**10 days recall**	36.7 (29.0, 45.1)	14.3 (6.8, 21.8)	< 0.0001*
**11 days recall**	39.6 (31.7, 48.0)	17.3 (9.3, 25.1)	< 0.0001*
**12 days recall**	42.5 (34.4, 50.9)	20.2 (12.4, 27.8)	< 0.0001*
**13 days recall**	39.6 (31.6, 48.0)	17.3 (9.3, 25.1)	< 0.0001*
**14 days recall**	38.13 (28.3, 44.4)	15.8 (7.8, 23.8)	< 0.0001*

*McNemar’s test *p* value < 0.05

*Reference - EBF percentage of 68% among infants 0–1 month 53.5% among 2–3 month and 22.3% among 4–5 months of age group

*short period recalls - single recall of the duration indicated in column 1

A significant overestimation was observed when short period recall was compared with the reference standard among infants of age 0–1 month, 2–3 months and 4–5 months. Using seven days recall the lowest overestimation of EBF was observed among the age category of 2–3 months and 4–5 months (Table 4).

**Fig. 1:**
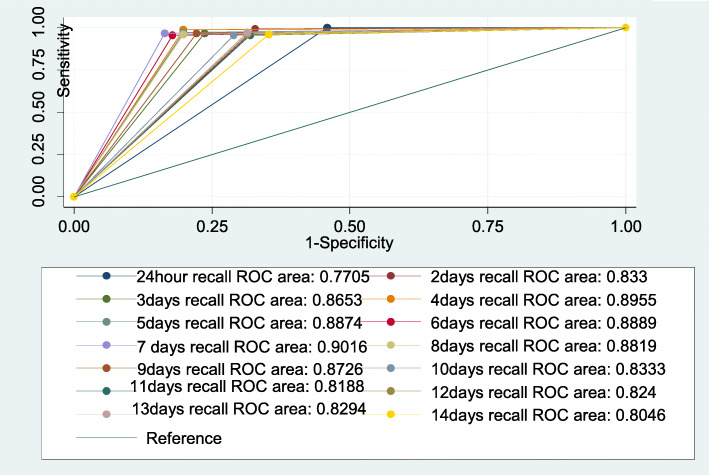
Sensitivity and specificity of short period recalls

The sensitivity and specificity of short recall methods was determined using the 14 repeated 24-h recall as a reference. The specificity of short period recalls ranged from 54.4 - 83.5%. Table 5 shows comparison of short period recalls with 14 repeated days 24-h recall as a reference in determining exclusive breastfeeding. The lowest specificity (54.4%) and positive predictive value (66.3%) was observed when single 24-h recall was compared against the reference standard. The highest specificity (83.5%) and positive predictive value (84.0%) was found when seven days recall was applied. The ability of the short period recalls to exclude those who were not exclusively breastfed (specificity) improved up to the recall over seven days period (Fig. 1).

**Table 5:** Comparison of short period recalls with 14 repeated days 24-h recall as a reference in determining exclusive breastfeeding, Butajira, Ethiopia, 2018

Test method	Sensitivity %	Specificity %	PPV %	NPV %	AUC
**24 h recall**	100 (98, 100)	54.4 (47.3, 61.3)	66.3 (60.4, 71.8)	100 (96.8, 100)	0.77 (0.74, 0.81)
**2 days recall**	99.5 (97, 100)	67.1 (60.3, 73.5)	72.9 (67.4, 78.7)	99.3 (96.1, 100)	0.833 (0.80, 0.86)
**3 days recall**	96.7 (93, 98.8)	76.3 (69.9, 81.9)	78.4 (72.5, 83.6)	96.3 (92.2, 98)	0.865 (0.83, 0.89)
**4 days recall**	98.9 (96.1, 99.9)	80.2 (74.1, 85.4)	81.6 (75.9, 86.5)	98.8 (95.8, 99.9)	0.896 (0.86, 0.92)
**5 days recall**	97.3 (93.8, 99.1)	80.2 (74.1, 85.4)	81.6 (75.6, 86.3)	97.1 (93.3, 99)	0.887 (0.86, 0.92)
**6 days recall**	95.7 (91.6, 98.1)	82.1 (76.2, 87.1)	82.6 (76.9, 87.5)	95.5 (91.3, 98)	0.889 (0.86, 0.91)
**7 days recall**	96.7 (93.0, 98.8)	83.5 (77.8, 88.3)	84.0 (78.3, 88.6)	96.6 (92.8, 98.8)	0.902 (0.87, 0.93)
**8 days recall**	96.2 (92.3, 98.5)	80.2 (74.1, 85.4)	81.2 (75.4, 86.2)	96.0 (91.8, 98.4)	0.882 (0.85, 0.91)
**9 days recall**	96.7 (93.0, 98.8)	77.8 (71.5, 83.2)	79.5 (73.6, 84.6)	96.4 (92.3, 98.7)	0.873 (0.84, 0.90)
**10 days recall**	95.7 (91.6, 98.1)	71.0 (64.3, 77.1)	74.6 (68.5, 80.0)	94.8 (90.1, 97.7)	0.833 (0.79, 0.87)
**11 days recall**	95.7 (91.6, 98.1)	68.1 (61.3, 74.4)	72.7 (66.7, 78.2)	94.6 (89.7, 97.7)	0.819 (0.78, 0.85)
**12 days recall**	96.2 (92.3, 98.5)	68.6 (61.8, 74.9)	73.1 (67.1, 78.6)	95.3 (90.6, 98.1)	0.824 (0.79, 0.86)
**13 days recall**	97.3 (93.8, 99.1)	68.6 (61.8, 74.9)	73.4 (67.3, 78.8)	96.6 (92.2, 98.9)	0.829 (0.79, 0.86)
**14 days recall**	96.2 (92.3, 98.5)	64.7 (57.8, 71.2)	70.8 (64.7, 76.4)	95.0 (90.0, 98.0)	0.805 (0.77, 0.84)

PPV-Positive Predictive Value NPV- Negative Predictive Value AUR- Area Under roc Curve

Sensitivity and specificity of short period recalls was also examined across the different age groups. The specificity in all age groups except the first group increased as the number of recall days increased up until the 7th day and decreased afterwards. The specificity increased from 37.5% obtained by 24-h recall to 77.5% obtained by six days recall among infants of age 0–1 month. Similarly, it increased from 39.0% obtained by 24-h recall to 81.4% obtained by seven days recall among infants age 2–3 months. This improvement in specificity was also observed among infants age 4–5 months.

The specificity, sensitivity and positive predictive value (PPV) of seven-day recall was examined against different sociodemographic variables. The specificity of seven days’ recall was higher (88.9%) among infants of age of 4–5 month and lower (72.5%) among 0–1 month old. Higher specificity of seven days recall was observed among mothers who attended school (86.5%). The variability was also observed among housewife and working mothers with specificity of 90 and 81.9% respectively.

## Discussion

This study aimed to assess an alternative indicator for EBF prevalence that addresses the trade-off between accuracy and feasibility of the measurement. We tested the accuracy of EBF prevalence estimated using recall over a one-week period. In doing so we tested the accuracy of estimates from varying recall periods as well. The exclusive breastfeeding prevalence estimated using the single 24-h recall was 71.4% (95% CI 66.9, 75.9) while it was 47.1% (95% CI 42.1, 52.1) when estimated by the reference method resulting in a 24.3% overestimation. A week recall (a recall over seven days’ period), resulted in the smallest discrepancy in EBF prevalence estimate (7.1%) as compared to the reference method.

Our findings indicated that 24-h recall over estimates exclusive breastfeeding prevalence. Such overestimations from 24-h recall have also been demonstrated in other studies [18, 19]. Similarly, former prospective studies identified an over estimation ranging 8.4 to 14.6% when estimates of 24-h recall method was compared with recall since birth [18–20]. Misclassification is common when single 24-h recall is used and this is misleading and undermines the effort to further improve breastfeeding practices.

Short periods recall methods showed a significant improvement in performance of estimating EBF practice. Comparing with the reference method, short period recalls showed improvement in specificity and PPV. For instance, there was improvement in specificity from 54.4% obtained from 24-h recall to 67.1% from a recall over two days period. Even though two days (48 h) recall had a better specificity and PPV, there were children who were still misclassified as being exclusively breastfed. This might be due to children who were fed additional food and drinks after the second day. This shows even though two days recall is better than 24-h recall in estimating breastfeeding practice, it still doesn’t capture the true change in feeding patterns. Several previous studies have also showed disagreement between different methods of recall. A study conducted in South Africa comparing 48 h recall with data collected prospectively at three data points 48 h recall methods similarly found low specificity (65–89%) and positive predictive value (31–48%) of 48 h recall method [21].

In our result, we have seen that EBF prevalence estimates decreased as the number of days the recall period spans progressively increased from one day to seven days. In addition, the ability of the short period recall methods in representing the feeding pattern improved up to a recall period that spanned for seven days period (a week recall). Among the multiple short periods of recall, a week recall had optimal specificity (83.5%) and positive predictive value (84.0%) promising a better ability to capture typical child feeding behavior. A study done to validate different methods of data collection on duration of exclusive breastfeeding has found similar results from a recall period spanning seven days [21]. Comparing with three times a week recall, a study has found seven day recall to accurately reflect EBF practices with 96% sensitivity and 94% specificity [21]. The increase in the number of days involved in the recall period, beyond a mere 24-h, might have captured more variability in child feeding practices since infant feeding practices vary widely within short periods of time [22].

In addition, week recall gives a more accurate picture of the usual infants feeding practice by capturing the day-to-day variation since it involves all days of the week. For example, in our study area there is a culture of giving infants an herb called “Anita” twice a week believing it would help the infant to gain weight. This plant is given on Wednesdays and Saturdays, which are market days. Such practices that have an impact on infant feeding practice cannot be captured by using a single day measurement, but by involving a longer recall period. Using a week recall method would help us capture such variations in feeding practices. In addition, since the recall period/duration is relatively short, mothers tend to have a better recall of what they have given thereby minimizing the possibility of recall bias.

Even though we have observed an improvement in the EBF estimate with short period recalls, we should be careful when we extend the recall period beyond seven days. We have observed a persistent increment in EBF over estimation when we asked a mother to recall a feeding practice beyond a week. In addition, the specificity and PPV also drops as the recall period extends from 8 to 14 days ranging (80.2–64.7%) and (81.2–70.8%) respectively. The presence of more cases of exclusive breastfeeding in the recall days exceeding 7th day, might be due to the presence of more false positive cases of EBF since the positive predictive value drops as the recall duration increases. As the number of days of recall increased beyond a week period, it is likely that women forget their feeding practices of the earlier days, and participants could have been misclassified as exclusively breastfed though that was not the case [6]. Ethiopia saw a rapid primary healthcare expansion in the last few decades. This promoted access of rural and peri urban communities to quality health information including optimal child feeding practices [23]. We presume most of our study participants have the information. This could have caused a desirability bias into denying introducing additional food in the first six months, a practice that they were taught was wrong, increasing the EBF prevalence.

We found out that 24-h recall does not capture the actual breastfeeding practices, at least in a small sample. It is intelligible that prospective studies are likely to be more accurate since it helps us to see infants moving in and out of the feeding categories [24]. A study done comparing single 24 h recalls with seven observation days has found discrepancy on the feeding pattern stating the possibility of capturing the day to day variation increased as the observation of days increase [12]. The consecutive days data help us to see the real picture of infants feeding history in order to categorize exclusive breastfeeding status. However, as longitudinal studies are resource intensive it may not meet the requirements of programmatic evaluations.

Infant feeding patterns are complex as an infant can be exclusively breastfed for a period, receive other food due to a change in circumstances, and then return to exclusive breastfeeding again. This complexity can only be captured either by repeated measurements or by employing a longer recall period. In order to overcome the challenge with overestimation from 24-h recall studies suggest using of recall since birth in addition to 24-h recall [18, 24]. A study exploring methods of measuring exclusive breastfeeding suggested the best approach would be to report indicators based on both point-in-time and life-long data [24]. On the other hand a longitudinal prospective study proposed adding EBF since birth as an indicator since indicators based on 24-h recall period could be inadequate and misleading for many reasons [13]. There are also different studies suggesting the use of recall since birth as an alternative method of assessment for EBF practice [18, 24]. Even though recall since birth determines the feeding practice throughout the infant’s life, there is high possibility of recall bias. However, we can certainly minimize the recall bias if we used a shorter recall period [24]. A study done in South Africa recommended the use of recall no longer than seven days to assess the prevalence EBF practice [21]. Our finding also indicated short recall periods can be an alternative method. However, these recall periods should not be greater than seven days (week recall) since the recall bias increased as we extended the recall period beyond the first seven days. Therefore, by taking improved specificity and feasibility into consideration we recommend a recall over a one-week period as it gives a more accurate estimate of the actual practice.

The major limitation of this study was the high possibility of desirability bias secondary to conducting repeated 24-h recalls. Though we could not completely avoid its effect we tried to minimize it by explaining the objective of the study and changing data collectors each day in order to give a fresh start for interviews every day. Another limitation of this study is not identifying potential confounding factors that might have influence on the practice. The strength of this study is taking repeated measure for consecutive days and itemized check list to aid the short period recalls method and minimize recall bias.

## Conclusions

A significant difference in exclusive breastfeeding estimate was observed among the different recall periods used. A week recall gave a comparable estimate to the reference method with optimal specificity and sensitivity. The recall period that is longer than 24-h helps to better capture variability in feeding practices and this point in time measurement optimizes the use of resources. We recommend for future efforts to report EBF using longer recall of 7 days. In addition, researches could also investigate factors that influence exclusive breastfeeding practice using longer recall.

## Data Availability

Data sets used and/or analyzed during the current study is available from the corresponding author on reasonable request.

## Abbreviation

EBF: Exclusive Breastfeeding
EDHS: Ethiopian Demographic Health Survey
HDSS: Health and Demographic Surveillance Site
IYCF: Infant and Young Child Feeding
NPV: Negative Predictive Value
ORS: Oral Rehydration Salt
PPV: Positive Predictive Value
WHO: World Health Organization
